# The Probiotic *Lactobacillus sakei* Subsp. *Sakei* and Hawthorn Extract Supplements Improved Growth Performance, Digestive Enzymes, Immunity, and Resistance to the Pesticide Acetamiprid in Common Carp (*Cyprinus carpio*)

**DOI:** 10.1155/2023/8506738

**Published:** 2023-03-06

**Authors:** Abdul-Hassan Mahdi Salih, Indrajit Patra, Ramaswamy Sivaraman, Rahim Alhamzawi, Kakhor M. Khalikov, Zahraa Haleem Al-qaim, Sahar Golgouneh, Mohammed Abed Jawad, Ali Hussein Adhab, Andrés Leonardo Vázquez-Cárdenas, Safoura Abarghouei

**Affiliations:** ^1^Department of Physiology, College of Medicine, University of Thi-Qar, Nasiriyah, Iraq; ^2^NIT Durgapur, West Bengal, India; ^3^Institution of Dwaraka Doss Goverdhan Doss Vaishnav College, Arumbakkam, University of Madras, Chennai, India; ^4^College of Administration and Economics, University of Al-Qadisiyah, Al Diwaniyah, Iraq; ^5^Department of Biological Chemistry, Samarkand State Medical University, Samarkand, Uzbekistan; ^6^Department of Anesthesia Techniques, Al-Mustaqbal University College, Iraq; ^7^Department of Fisheries, Faculty of Natural Resources, University of Tehran, Tehran, Iran; ^8^Al-Nisour University College, Baghdad, Iraq; ^9^Department of Medical Laboratory Technics, Al-Zahrawi University College, Karbala, Iraq; ^10^Carrera de Enfermería de la Universidad Católica de Cuenca Sede Azogues, Azogues, Ecuador; ^11^Baharavaran Nastaran Agricultural Applied Scientific Training Center, Applied Scientific University, Qom, Iran

## Abstract

This study evaluated the impacts of the probiotic, *Lactobacillus sakei* (*L. sakei*), and the extract of hawthorn, *Crataegus elbursensis*, on growth and immunity of the common carp exposed to acetamiprid. Fish (mean ± SE: 11.48 ± 0.1 g) feeding was done with formulated diets (*T*_1_ (control): no supplementation, *T*_2_: 1 × 10^6^ CFU/g LS (*Lactobacillus sakei*), T3: 1 × 10^8^ CFU/g LS, *T*_4_: 0.5% hawthorn extract (HWE), and *T*_5_: 1% HWE) for 60 days and then exposed to acetamiprid for 14 days. The growth performance improved in the fish fed LS at dietary level of 1 × 10^8^ CFU/g, even after exposure to acetamiprid (*P* < 0.05). Intestinal *Lactobacillus sakei* (CFU/g) load increased (*P* < 0.05), following supplementation with the probiotic-enriched diet. The LS-treated fish had increases in the activity of digestive enzymes (*P* < 0.05). Both LS and HWE stimulated antioxidant enzymes and immune system components in serum and mucus (alkaline phosphatase (ALP), protease, total Ig, and lysozyme) (*P* < 0.05). However, the changes were different depending on the kind of the supplement. The malondialdehyde (MDA) levels decreased in HWE-treated fish after acetamiprid exposure (*P* < 0.05). Both LS and HWE reduced the liver metabolic enzymes (LDH, ALP, AST, ALT, and LDH) in serum both before and after exposure to the pesticide (*P* < 0.05). However, each enzyme exhibited a different change trend depending on the type of the supplement. HWE showed a stress-ameliorating effect, as glucose and cortisol levels declined in the HWE-treated fish (*P* < 0.05). This study indicated the immunomodulatory impacts of LS (1 × 10^8^ CFU/g) and HWE (at dietary levels of 0.5–1%). The probiotic showed more performance compared to HWE. However, the HWE mitigated oxidative stress more efficiently than the probiotic.

## 1. Introduction

Today, the development of agriculture has been with widespread use of pesticides (PCs) to control pests [1]. PCs enter aquatic ecosystems through various ways, including field drainage and runoff from land, and adversely affect aquatic life including fish [2]. Since the use of PCs is an unavoidable issue, we must look for solutions to minimize the harmful impacts of these chemicals on aquatic organisms. Probiotics are applied in fish culture as dietary supplements for various purposes. It is a real fact that probiotics improve growth, digestibility, immune system, and resistance to diseases in fish [3–5]. Furthermore, it is reported that probiotics may be useful in ameliorating the toxicity induced by PCs [6–8]. However, this role is rarely studied in fish [9].

In addition to probiotics, herbal supplements and their compounds are known to enhance fish immunity [10–12] and reduce the toxic effects of PCs ([13, 14][15, 16]). However, these studies are few and we need to develop our knowledge about herbs and their role as toxin-ameliorating agent in fish. The immune and antioxidant-stimulating properties of medicinal herbs mainly return to a group of compounds such as flavonoids, carotenoids, alkaloids, tannins, lectins, terpenoids, and polyphenols in their biochemical composition [17–19].

The hawthorn is a species of the family Rosaceae that is used in traditional medicine for a long time [20]. In traditional medicine, hawthorn is used to treat digestive disruptions, blood stasis, hypertension, hyperlipidemia, amenorrhea, insomnia, arthritis, and muscle pains [21]. In addition, many studies have reported a variety of functions for hawthorn including antimicrobial, antioxidant, anti-inflammatory, and liver protective activities [22–24]. The biochemical composition of hawthorn fruit includes various phytochemicals such as phenolic acids, flavonoids, proanthocyanins, essential oils, and aromatic amines [23]. In fish, a few studies have used hawthorn in diet to improve the cellular and humoral immunity. In challenge with *Vibrio harveyi*, better immunity is obtained in hawthorn-treated golden pompano, *Trachinotus ovatus* [21]. Hawthorn, *Crataegus mexicana*, improved antioxidant and immune system in *Longfin yellowtail* and *Seriola rivoliana* [25]. Literatures have showed no study about protective effects of hawthorn against pesticides. Acetamiprid is a neonicotinoid insecticide, which is widely applied in agriculture for control of pests throughout the world [26, 27]. The aim of the present study was to examine the potentials of the hawthorn extract and the probiotic bacteria *Lactobacillus sakei* on growth performance, immune and antioxidant potentials, and resistance to acetamiprid toxicity in the common carp, *Cyprinus carpio*.

## 2. Materials and Methods

### 2.1. Probiotic Preparations

The probiotic *Lactobacillus sakei* subsp. *sakei* 15521 was prepared from the Iranian Research Organization for Science and Technology in Tehran, Iran, as lyophilized form and incubated in Rogosa and Sharpe agar (MRS) culture medium at 38°C for 48 h. Then, the medium was centrifuged for at 4°C (×4000*g*) 4 min and the supernatant discarded. The pellets were washed three times using phosphate buffer, bacteria added to the phosphate buffer again, and the experimental concentrations were determined at 600 nm by a spectrophotometer. Finally, the experimental concentrations of bacteria were added to the basic food [28].

### 2.2. Hawthorn Extract

The fruits of hawthorn were provided from Shast Kalate forest, Gorgan, Iran. The fruits were dried at 40°C in an oven. 200 g of dried hawthorn powder was added to 80% ethanol and stirred in an incubator with shaking for 24 h. After that, the suspended particles were removed using Whatman No. 1 paper. The extract was concentrated by a rotary evaporator at 40°C, pulverized by freeze drying, and stored at −18°C until use.

### 2.3. Antioxidant Power of the Extract

The antioxidant power of the extract was evaluated by four methods as follows and the results presented in Table 1.

#### 2.3.1. Antioxidant Activity by Free Radical Scavenging (DPPH) Method

100 *μ*l of the extract was mixed with 0.2 ml of 0.1 M 2,2-diphenyl-1-picrylhydrazyl (DPPH) in 150 *μ*M methanol, incubated at 22°C for 35 min, and then its absorbance read at 520 nm. Antioxidant activity was finally calculated using the following formula [29]:

(1) $$\begin{array}{c} \%\text{inhibition}=100\left(A_{\text{blank}}-\frac{A_{\text{sample}}}{A_{\text{blank}}}\right). \end{array}$$

#### 2.3.2. Total Phenol Assay

Total phenolic evaluation was performed using the Folin–Ciocalteu method. Briefly, 0.12 ml of the extract was mixed with 0.05 ml of 10% Folin–Ciocalteu reagent. 30 *μ*l of 20% saturated sodium carbonate solution was added to the solution, incubated for 1 h at 37°C, and absorbance was read at 735 nm after 10 min of storage under dark condition [30].

#### 2.3.3. Total Flavonoid Assay

The total flavonoid content of the hawthorn extract was assayed at 510 nm using aluminum chloride method [31]. A mixture of 250 *μ*l of the extract, 1250 *μ*l of distilled water, and 75 *μ*l of sodium nitrate solution (5%) was prepared, and then, aluminum chloride (10%) was added upon 5 min incubation at 23°C. After incubation, a solution of sodium hydroxide (500 *μ*l) and distilled water (775 *μ*l) was prepared and added to the solution and homogenized, and the adsorption spectrum was read.

#### 2.3.4. Evaluation of Total Antioxidant Capacity (TAC)

To assay TAC, the extract solution (0.1 ml) and 1 ml of the reagent (0.6 M sulfuric acid + 28 mM sodium phosphate + 4 mM ammonium moly date) were poured into a tube and sealed for 1.5 h in water bath at 95°C. After 5 min, the absorbance was read at 695 nm [32].

### 2.4. Experimental Diets

To prepare experimental diets, firstly a commercial feed (protein: 34%, fat: 6%, fiber: 5%, moisture: 8%, ash: 9%, and phosphorus: 1%) was purchased as basal diet from Faradaneh company, Iran, which did not contain any supplements. The basal diet was thoroughly ground, mixed with some water to make dough, pelleted by grinder, and dried at 35°C. The experimental diets were prepared by adding *Lactobacillus sakei* at concentrations 1 × 10^6^ and 1 × 10^8^ CFU/g feed and hawthorn extract at levels of 0.5 and 1% according to Tan et al. [21]. Doses were selected based on positive results from previous reports on the growth and health of other aquatic animals [21, 33, 34].

### 2.5. Fish and Experimental Procedure

650 common carps (8.2 ± 0.24 g; mean ± SE) were provided from a local farm in Khuzestan province (Shushtar city, Iran) and transferred to a local farm in Tehran. The specimens were stocked in 1000 l tanks and acclimatized for 2 weeks with culture condition (temperature: 25 ± 0.6°C, dissolved oxygen: 6.8 ± 0.5 mg/l, pH: 7.4 ± 0.3, nonionized ammonia: 0.04 ± 0.03). During acclimation period, fish were fed basal diet 3 times a day (2.5% of body weight). After acclimation period, fish (*n* = 600) (11.48 ± 0.1 g; mean ± SE) were distributed into 15 tanks (40 fish/tank) as four experimental treatments (*T*_2_: fish fed diet containing 1 × 10^6^ probiotic, *T*_3_: fish fed diet containing 1 × 10^8^ probiotic, *T*_4_: fish fed diet containing 0.5% hawthorn extract, *T*_5_: fish fed diet containing 1% hawthorn extract) and one control group (*T*_1_ (control): nonsupplemented fish) in three replicates. Fish were fed experimental diets at a feeding rate of 2.5% of body weight for 60 days [35].

### 2.6. Acetamiprid and Exposure Trial

Before exposure test, the lethal and acute dosages of acetamiprid for the fish were determined to select experimental concentrations. To estimate lethal range of acetamiprid, fish (*n* = 30, 10/tank) were exposed to dosages of 0, 6, 8, 10, 12, 14, 16, and 18 mg/l of acetamiprid for 96 h to estimate the LC_50_ [36]. The mortality of the fish was recorded upon exposure with time at 24, 48, 72, and 96 h. The probit statistical analysis was used to estimate the lethal concentrations (Table 2) inducing 10% (LC_10_), 30% (LC_30_), 50% (LC_50_), and 70% (LC_70_) mortality.

After feeding period, fish were exposed to acetamiprid at a concentration of 25% of LC_50_ for 14 days [16]. The static renewal design was used with the daily water change of 70%.

### 2.7. Growth Indices

After the feeding experiment, feeding was stopped for 24 h and fish were anesthetized using 100 mg/l eugenol. Growth and nutrition indices were calculated by sampling all fish per tank using the following formulas [35]:

(2) $$\begin{array}{c} \text{weight gain }\left(\text{WG},\text{g}\right)=\text{Fiw}-\text{Iwi}, \end{array}$$

where Fiw is the final weight and Iwi is the initial weight.

(3) $$\begin{array}{c} \text{Specific growth rate }\left(\text{SGR},\%/\text{day}\right)=100\times \left[\left(\ln\text{ Fiw}-\ln/\text{days}\right)\right],\\ \text{Feed conversion ratio},\\ \text{Protein efficiency ratio},\\ \text{Survival rate }\left(\text{SR},\%\right)=\left(\text{Alive fish number}/\text{total fish number}\right)\times 100. \end{array}$$

### 2.8. Sampling

The blood and mucus samples were taken after feeding period and after 14 days of exposure to acetamiprid.

### 2.9. Digestive Enzyme Activity

To determine digestive enzyme activities, fish (*n* = 3/tank) were randomly sampled, euthanized using high dosage of eugenol, dissected, and after the intestine tissue separated. The intestine was emptied and weighed and then homogenized mechanically using Tris buffer (Heidolph® SilentCrusher-M, Heidolph, Nuremberg, Germany) [37]. The supernatant was obtained by centrifugation at 4°C (×6000*g* for 10 min) and stored at −80°C. Amylase was estimated at 600 nm upon reaction of the enzyme with 2% starch as substrate [38]. Lipase was assayed at 405 nm upon the action of the enzyme on polyphenol myristate, as target [39]. Protease enzyme was measured at 440 nm by García-Carreño [40] method. Azo-casein was used as target for the enzyme.

### 2.10. Intestinal Microbial Population

After disinfecting of the skin by 70% ethanol, the fish abdominal cavity was dissected and the intestine separated, washed, and homogenized in phosphate buffer (PBS, pH = 7.2) using a tissue homogenizer. The homogenized solution was diluted in phosphate buffer. The bacterial colonies were grown on MRS (Merck, Germany) and TSA medium at 30°C for 48 h to assay lactic acid bacteria (LAB) and total intestinal bacteria (TBC), respectively [41].

### 2.11. Immunological Assays

To determine immune parameters of serum, fish (*n* = 3/tank) were anesthetized by eugenol (90 mg/l), and blood was taken from caudal vein using a 1.5 ml syringe, stored in heparinized tube, left at 23°C for 90 min h, and centrifuged at 4°C (3500 × *g*, 8 min) to collect serum. The serum was stored at −75°C for further assays. Mucus sampling was done by putting fish in polyethylene bags containing saline solution. The supernatant was separated after 3 min through centrifuging of the mucus at 4°C (2650 × *g* for 12 min) [42].

Serum and mucosal lysozyme activity was measured at 550 nm according to Mirghaed et al. [43] method based on the ability of serum or mucus in lysis of *Micrococcus luteus*.

Complement activity was measured using sheep red blood cells [44]. 500 *μ*l of serum sample was diluted sequentially (pH = 7) using veronal buffer (EGTA + gelatin + magnesium, pH = 7). 200 *μ*l of red blood cell suspension was added to each tube. The tubes were incubated for 15 min at 15°C. Hemolysis was stopped by adding 10 mmol gelatin veronal buffer−EDTA. After centrifugation, the amount of hemolysis was measured in supernatant at 414 nm.

Total Ig content was calculated based on Siwicki [45] method through calculating the difference between protein content of serum and mucus before and after precipitating by 12% polyethylene glycol. The activity of myeloperoxidase (MPO) was estimated at 450 nm by a microplate reader upon reaction of the enzyme with tetramethylbenzidine hydrochloride as target [46]. Nitroblue tetrazolium (NBT) reduction was assayed at 540 nm upon reaction of the samples with N,N-dimethylformamide [47]. The protease activity was assayed at 450 nm upon reaction of the enzyme with azo-casein (100 mM) as target at 30°C for 20 h [48].

### 2.12. Biochemical and Enzymatic Assays

The antioxidant potentials were evaluated by estimating glutathione peroxidase (GPx), catalase (CAT), and superoxide dismutase (SOD) using assay kits (Zellbio, Berlin, Germany) and manufacturers' instructions. SOD was assayed upon reduction of cytochrome C [49]. CAT was estimated upon decomposition rate of hydrogen peroxide [50]. Malondialdehyde (MDA) as an indicator of lipid peroxidation was measured based on its reaction with thiobarbituric acid (TBARS) [51].

Liver enzymes were assayed using commercial kits (Pars Azmun Co., Tehran, Iran) for ALP, AST, and ALT according to the manufacturer's protocol [52].

An ELISA method was applied to assay cortisol levels using an assay kit (IBL Co., Germany). Glucose changes were also measured by Pars Azmun commercial kit, Iran [52]. Total protein in serum was measured by the Bradford [53] method. Also, the albumin concentration was estimated by colorimetric method using the Nicholson method at 620 nm [54]. Globulin was assayed by calculation of the difference of protein and albumin content in blood. The activity of alkaline phosphatase activity in mucus was measured by the Pars Azmun commercial kit, Iran, at 405 nm according to manufacturer's instructions [55].

### 2.13. Data Analysis

The data (mean ± SE) was analysed by version 16 of SPSS software. After normality test by the Kolmogorov*-*Smirnov test, the differences among the means were evaluated by one-way analysis of variance, followed by the comparison of the means by Tukey test.

## 3. Results

### 3.1. Growth Parameters

The results of fish growth are presented in Table 3. The final weight (FW) and WG increased (*P* < 0.05) in fish of 1 × 10^6^ (*T*_2_) and 1 × 10^8^ (*T*_3_) in comparison with nontreated fish. SGR had similar values (*P* > 0.05) among nontreated fish, 1 × 10^6^ probiotic and hawthorn extract treatments. The FCR values with the lowest value in *T*_3_ were lower in *T*_2_ and *T*_3_ than in nontreated fish and other treatments (*P* < 0.05). FCR values had no differences between fish 0.5% and 1% hawthorn extract (*P* > 0.05). SR values exhibited no differences among control and other experimental groups (*P* > 0.05).

After exposure to acetamiprid, FW values of *T*_2_, *T*_3_, and *T*_4_ had higher values in comparison with nontreated fish (*P* < 0.05). No differences were found in FW between control and *T*_5_ (*P* > 0.05). WG values in fish of 1 × 10^6^ and 1 × 10^8^ probiotic were higher than those in control (*P* < 0.05). FCR values with the lowest value in *T*_3_ were lower in fish of 1 × 10^6^ and 1 × 10^8^ probiotic than in control and other treatments (*P* < 0.05). FCR values had clear decreases in all supplemented treatments in comparison with control (*P* < 0.05). Treatment *T*_3_ had lower FCR in comparison with others (*P* < 0.05). SR values had no differences (*P* > 0.05) in all experimental groups.

### 3.2. Digestive Enzymes

Digestive enzyme activity of the groups is presented in Table 4. Protease and amylase activities in the probiotic treatments increased in comparison with control (*P* < 0.05). Similar values were found in protease and amylase activities of control and fish of 0.5% and 1% hawthorn extract (*P* > 0.05). Lipase activity had no differences (*P* > 0.05) among all experimental groups after the feeding period.

### 3.3. Bacterial Load of the Intestine

The concentration of *Lactobacillus sakei* (CFU/g) in intestine tissue considerably increased in *T*_2_ and *T*_3_ in comparison with control and *T*_4_ and *T*_5_ (supplementary file (available here), *P* < 0.05). Bacterial concentration of control had no differences with those in fish of 0.5% and 1% hawthorn extract (supplementary file, *P* > 0.05). Total bacterial concentration (TBC) in the intestine also exhibited no differences (*P* < 0.05) in all groups (supplementary file).

### 3.4. Serum Immune Parameters

The results of the immune parameters are presented in Table 5. The lysozyme activity elevated in the supplemented groups than in control (*P* < 0.05). Lysozyme activity had similar values (*P* > 0.05) among all supplemented groups. The Ig levels and ACH_50_ activity had no differences (*P* > 0.05) in all groups. MPO activity increased (*P* < 0.05) in probiotic treatments and 0.5% hawthorn extract in comparison with control. The maximum MPO was observed in *T*_3_ (*P* < 0.05). NBT activity increased in the probiotic supplemented fish in comparison with control (*P* < 0.05). Protease activity showed significant increases (*P* < 0.05) in *T*_5_ in comparison with control, while the enzyme activity of control had no differences with other treatments (*P* > 0.05).

After exposure to acetamiprid, the lysozyme exhibited more activity (*P* < 0.05) in all treatments than in control. Lysozyme showed similar values (*P* > 0.05) in supplemented fish. ACH_50_ elevated (*P* < 0.05) in *T*_4_ and *T*_5_ in comparison with control. ACH_50_ activity had no differences (*P* > 0.05) among nontreated and probiotic-treated fish. The Ig was higher (*P* < 0.05) in fish of 1 × 10^8^ probiotic and 5% hawthorn extract than in nontreated fish. MPO activity raised in fish of 1 × 10^8^ probiotic and 1% hawthorn extract (*P* < 0.05), while it had no differences in supplemented fish (*P* > 0.05). NBT raised in *T*_3_ in comparison with control (*P* < 0.05).

### 3.5. Immune Parameters of Mucus

**Figure 1:**
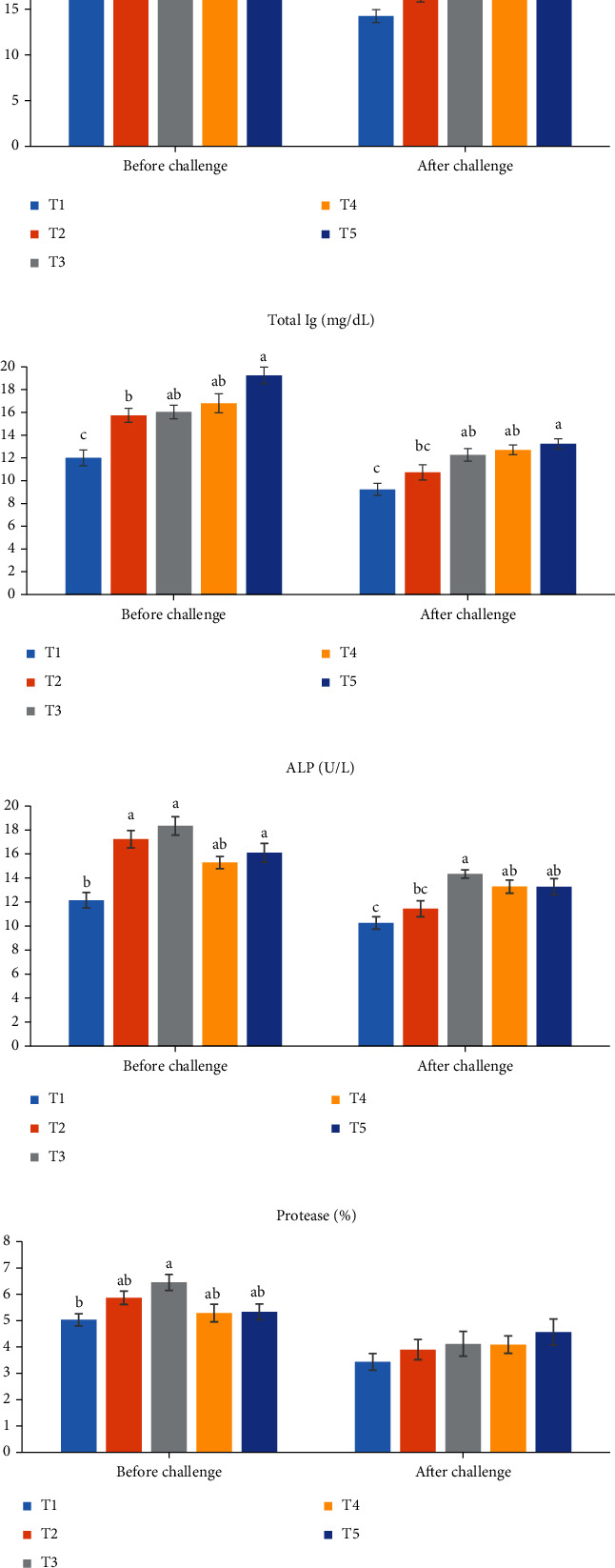
The mucus immune parameters in common carp, *Cyprinus carpio*, after 60 days of supplementation with experimental diets: *T*_1_ (control): nonsupplemented fish; *T*_2_: fish fed diet containing 1 × 10^6^ probiotic; *T*_3_: fish fed diet containing 1 × 10^8^ probiotic; *T*_4_: fish fed diet containing 0.5% hawthorn extract; *T*_5_: fish fed diet containing 1% hawthorn extract. Total Ig: total immunoglobulin; ALP: alkaline phosphatase activity. Data represented as mean ± SE. Different letters in the same row indicate significant differences (*P* < 0.05).

The mucosal lysozyme elevated in *T*_3_ and *T*_5_ in comparison with control after the feeding period (Figure 1(a), *P* < 0.05). Similar activity was observed for lysozyme in all supplemented groups (Figure 1(a), *P* > 0.05). The Ig levels with maximum levels in *T*_5_ elevated in all supplemented fish (Figure 1(b), *P* > 0.05), while it showed similar values (*P* > 0.05) among fish of 1 × 10^8^ probiotic and hawthorn extract treatments (Figure 1(b)). ALP (Figure 1(c)) increased (*P* < 0.05) in the probiotic and 1% hawthorn extract treatment in comparison with control. ALP had similar values in all supplemented fish (Figure 1(c), *P* > 0.05). Protease raised in *T*_3_ compared to control (Figure 1(d), *P* < 0.05). Protease exhibited similar activity in others (Figure 1(d), *P* > 0.05).

After exposure to acetamiprid, the lysozyme (Figure 1(a)) and ALP (Figure 1(c)) activities and Ig (Figure 1(b)) levels elevated (*P* < 0.05) in *T*_3_, *T*_4_, and *T*_5_ in comparison with control, while those had similar values (*P* > 0.05) in control and *T*_2_ (Figures 1(a)–1(c)). The enzyme protease activity was similar (*P* > 0.05) for all groups (Figure 1(d)).

### 3.6. Blood Biochemicals

The results of the liver metabolic enzymes are presented in Table 6. Liver metabolic enzymes showed significant changes in serum in the treatments after feeding period (*P* < 0.05). AST and ALT activities declined in *T*_5_ in comparison with control (*P* < 0.05). In addition, AST and ALT showed similar activities among all supplemented groups (*P* > 0.05). ALP activity had no differences among all treatments (*P* > 0.05). LDH activity decreased (*P* < 0.05) in *T*_3_, *T*_4_, and *T*_5_ in comparison with control and *T*_2_. The lowest LDH activity (*P* < 0.05) was related to *T*_5_.

The cortisol levels (Table 7, *P* < 0.05) declined in *T*_4_ and *T*_5_ in comparison with nontreated fish. Cortisol levels in control showed similar levels with fish of 1 × 10^6^ and 1 × 10^8^ probiotic (*P* > 0.05). Glucose had similar values (*P* > 0.05) among experimental groups (Table 7). The globulin, total protein, and albumin levels increased (*P* < 0.05) in *T*_4_ and *T*_5_ in comparison with control (Table 7). Total protein, albumin, and globulin content of control had no differences (*P* < 0.05) with *T*_2_ and *T*_3_ (Table 7, *P* > 0.05). The highest levels of total protein, globulin, and albumin were found in *T*_4_ (Table 7).

The MDA (Table 8) levels and CAT activity (Table 8) showed no changes among all groups (*P* > 0.05). SOD activity (Table 8) raised (*P* < 0.05) in *T*_3_, *T*_4_, and *T*_5_ in comparison with control. Maximum SOD (*P* < 0.05) was observed in *T*_5_. GPx (Table 8) was higher in *T*_5_ than in control (*P* < 0.05).

After exposure to acetamiprid (Table 6), the ALT activity decreased in *T*_5_ in comparison with control (*P* < 0.05). ALT activity exhibited similar values (*P* > 0.05) among all supplemented groups. ALP and AST activities decreased in *T*_3_, *T*_4_, and *T*_5_ in comparison with control (*P* < 0.05). The lowest ALP and AST activities were found in *T*_5_ (Table 6, *P* < 0.05). There were no differences in ALP and AST activities between control and *T*_2_ (*P* > 0.05). The LDH activity of all treatments decreased in comparison with control (*P* < 0.05). The lowest LDH activity (*P* < 0.05) was related to *T*_4_ and *T*_5_.

Cortisol (Table 7) decreased in *T*_3_, *T*_4_, and *T*_5_ in comparison with nontreated fish (*P* < 0.05). Cortisol concentrations had similar values in control and *T*_2_ (*P* > 0.05). Glucose (Table 7) declined in *T*_5_ in comparison with nontreated fish (*P* < 0.05). Total protein (Table 7) increased in *T*_4_ and *T*_5_ in comparison with nontreated fish (*P* < 0.05). Albumin and globulin (Table 7) content in control had similar values (*P* > 0.05) with the supplemented fish.

The MDA (Table 8) levels reduced (*P* < 0.05) in *T*_4_ and *T*_5_ in comparison with control. SOD activity packed (*P* < 0.05) in fish of 1 × 10^8^ probiotic and 0.5-1% hawthorn extract in comparison with control, with maximum activity in *T*_5_. GPx activity raised (*P* < 0.05) in hawthorn extract treatments in comparison with control. GPx in nontreated fish was similar to fish of 1 × 10^6^ and 1 × 10^8^ probiotic (*P* > 0.05). The CAT activity in treated fish showed no changes (*P* > 0.05) after exposure to acetamiprid.

## 4. Discussion

The application of probiotics and herbs has increased in aquaculture to improve fish growth and immunity. We investigated the prompting impacts of the probiotic, *Lactobacillus sakei*, and a medicinal plant, hawthorn extract (HWE), on growth, immunity, and the toxin resistance ability in the common carp. The growth results showed that the probiotic at high dietary level (1 × 10^8^ CFU/g) can effectively improve the growth performance (i.e., FW, WG, SGR, and FCR), even after exposure to acetamiprid, as the highest growth efficiency was related 1 × 10^8^ CFU/g probiotic. In addition, we observed increases in the intestinal load of lactic acid bacteria in the probiotic-supplemented fish in comparison with control, indicating the efficient modulation of intestinal bacterial flora by the dietary probiotic. By contrast, such effects were not observed in fish fed HWE (i.e., *T*_4_: 0.5% HWE and *T*_5_: 1% HWE). Therefore, these results indicate that the probiotic has more improving effect on growth compared to the plant extract. The enhancing effect of *L. sakei* on growth may return to the general role of probiotics in improving digestion and absorption of the nutrients, digestive enzyme activities, and production of growth-stimulant metabolites and in excluding the pathogenic bacteria in the gut, as previously shown in many researches [56, 57]. In this regard, we observed elevations in the activity of amylase and protease in response to dietary *L. sakei*. As the results showed, the use of the probiotic improved growth indices, even after exposure of the fish to acetamiprid. Although the role of probiotics in reducing the pesticide toxicity has been experimentally reported [6, 7, 58, 59], it is rarely studied in fish [9, 60]. The probiotic bacteria may break down pesticides over bioremediation process using the enzymes including phosphotriesterases, phosphatases, carboxylesterases, and organophosphate hydrolases to meet their needs to nitrogen, carbon, and energy [61, 62]. In addition, fermentation is another process that probiotic bacteria may use to metabolize pesticides [6]. Similarly, a combination of the probiotics (*Bacillus subtilis* + *Lactococcus lactis and L. lactis* + *Saccharomyces cerevisiae*) in diet of Indian carp *Labeo rohita* mitigated the retarded growth in fenvalerate-exposed fish, which was related to the prompting effects of the probiotic on food consumption [60]. In Java barb, *Barbonymus gonionotus*, the use of *Lactobacillus* spp. in diet reasonably restored the reduced growth in fish exposed to the pesticide *Sumithion* [9].

Many researches have shown the role of probiotics and herbs in improving the fish immune system [63–66]. The immune-stimulating properties of *Lactobacillus sakei* is also reported in fish [33, 34, 67]. The results of this study confirmed this immunogenic role, because we observed increases in immune components and also antioxidant enzymes in the supplemented fish; however, the change trends were different depending on the kind of the supplement. In this regard, fish fed diet containing 1 × 10^8^ probiotic and 0.5 and 1% hawthorn extract showed higher values of antioxidant and immune components in almost all treatments both before and after exposure to acetamiprid. However, hawthorn treatments seem to be more effective in improving the immune and antioxidant system than the probiotic treatments. Although the stimulating properties of probiotics on fish immunity are widely reported, there is little data about this function with pesticides [68]. Probiotics improve the immune system in a variety of ways, such as modulating of intestinal bacterial flora, competing with and eliminating pathogenic bacteria in the gut, stimulating the activity of innate immune system components such as lysozyme, complement, and immunoglobulin, and upregulation of immune-related gene expressions [65, 69–71]. Also, as mentioned earlier, probiotics can biodegradate and fermentate the pesticides in the gastrointestinal tract, which may reduce their immunotoxic impacts [6, 61, 62]. Although the role of probiotics as antioxidants and also their inducing effect on enzymatic antioxidant enzyme system are reported, its mechanism is still unknown. Antioxidant function of probiotics in fish is attributed to their prompting impacts on modulation of antioxidant genes. For example, in the gilthead seabream *Sparus aurata*, the expression of SOD and GPx in the mucus is upregulated in *Shewanella putrefaciens- and Bacillus-*treated fish [72]. Similarly, the SOD and GPx values were stimulated in response to dietary *B. licheniformis* in *O. mossambicus* [73]. In Indian carp, *Labeo rohita*, the diet containing *Bacillus subtilis* + *Lactococcus lactis and L. lactis* + *Saccharomyces cerevisiae* mitigated the toxic effects of the insecticide fenvalerate. In their study, the probiotic-containing diets had sparing effects on SOD and CAT activities. In addition, the probiotic improved NBT, total protein, and albumin values in blood of fenvalerate-exposed fish [60].

There were many studies reporting increases in MDA levels as the main marker of oxidative stress, following exposure to pesticides in fish [74, 75]. MDA levels declined in hawthorn extract-supplemented fish, which may suggest an ameliorating effect for the supplement on the oxidative stress. The mitigating role of medicinal plants on pesticide-induced oxidative stress has been also reported in other studies, which is usually attributed to their stimulating effects on antioxidant enzymes and the presence of some compounds such as phenolic compounds and flavonoids in their biochemical composition [16, 76, 77].

In blood, increased concentration of hepatic enzymes (ME) may indirectly reflect liver dysfunctions and damages, although ME are not generally specific [78]. The use of the probiotic and hawthorn reduced LME levels both before and after exposure to the pesticide. However, each enzyme showed different change trends depending on the type of the supplement. Decreased levels of LME may demonstrate a protective effect for the supplements on the liver [79]. In this regard, it seems that the use of 1% hawthorn has more performance than other supplements, because LME decreased in this treatment. Similarly, the reducing effects of probiotics [80] and herbs [77, 81, 82] on LME are previously reported in pesticide-treated fish.

As the main stress hormone, cortisol is released in blood after exposure to variety of stressors. Cortisol breaks down hepatic glycogen stores to release glucose into the bloodstream to meet energetic costs of the stress [83, 84]. In this study, hawthorn extract showed a stress-ameliorating effect, because cortisol and glucose levels reduced in HWE-treated fish both before and after exposure to pesticide. Pesticide-induced stress in fish and following increases in cortisol and glucose have been reported in many studies [85–87]. The current results were in line with previous studies that have reported the mitigating effect of herbs on stress caused by pesticides [13, 16, 88, 89]. In probiotic treatments, the cortisol levels decreased only in the treatment 1 × 10^8^ CFC/g probiotic after exposure to the pesticide, which may return to the mitigating impacts of probiotics on stress [90, 91]. In this study, although fish growth and immunity improved in the supplemented fish, the survival rate was not affected over exposure to the pesticide.

The outputs of this study revealed the immunomodulatory properties of LS (at dietary levels of 1 × 10^8^ CFU/g) and HWE (at dietary levels of 0.5-1%) in the common carp. The probiotic exhibited more growth-prompting effect compared to HWE. However, the HWE mitigated oxidative stress more efficiently compared to the probiotic.

## Figures and Tables

**Table 1:** Evaluation of the antioxidant power of hawthorn extract using different methods.

Assay methods	
Total phenolics (mg GAE/g)	65.2 ± 1.10
Total flavonoids (mg QE/g)	2.10 ± 0.11
DPPH % inhibition percentage	64.77 ± 2.20
Total antioxidant capacity (*μ*g/ml)	0.52 ± 0.08

**Table 2:** Lethal concentrations (LC_10-90_) of acetamiprid (Mospilan) over time (24-96 h) for *Cyprinus carpio.*

Point	Concentration (mg/l)							
	LC_50_ 24 h	Upper bound Lower bound	LC_50_ 48 h	Upper bound Lower bound	LC_50_ 72 h	Upper bound Lower bound	LC_50_ 96 h	Upper bound Lower bound
LC_10_	9.87	10.76 8.58	8.64	9.65 7.18	7.75	8.78 6.27	6.02	7.11 4.40
LC_30_	12.18	12.87 11.37	11.30	12.04 10.39	10.34	11.09 9.41	8.49	9.28 7.45
LC_50_	**13.78**	**14.52** **13.10**	**13.14**	**13.92** **12.40**	**12.13**	**12.87** **11.40**	**10.21**	**10.92** **9.44**
LC_70_	15.39	16.37 14.64	14.98	16.04 14.17	13.92	14.88 13.17	11.92	12.76 11.21
LC_90_	17.70	19.24 16.67	17.63	19.32 16.49	16.51	18.02 15.47	14.40	15.73 13.47

**Table 3:** The growth performance and survival rate in common carp, *Cyprinus carpio*, after 60 days of supplementation with experimental diets.

Status	Parameters	*T*_1_ (control)	*T*_2_	*T*_3_	*T*_4_	*T*_5_
Before challenge	IW (g)	11.53 ± 0.26	11.46 ± 0.29	11.36 ± 0.18	11.36 ± 0.26	11.66 ± 0.24
	FW (g)	42.70 ± 1.04*c*	48.00 ± 0.45^ab^	51.23 ± 0.88^a^	45.23 ± 0.67^bc^	43.46 ± 0.78^c^
	WG (g)	31.16 ± 0.90^c^	36.53 ± 0.74^ab^	39.86 ± 0.89^a^	33.86 ± 0.93^bc^	31.80 ± 0.70^c^
	FCR	1.98 ± 0.08^a^	1.70 ± 0.05^bc^	1.48 ± 0.04^c^	1.79 ± 0.03^ab^	1.93 ± 0.05^ab^
	SGR (%d^−1^)	2.18 ± 0.03^b^	2.43 ± 0.10^ab^	2.58 ± 0.10^a^	2.30 ± 0.06^ab^	2.19 ± 0.03^b^
	SR (%)	95.33 ± 2.33	96.66 ± 2.02	97.66 ± 2.33	96.66 ± 1.66	95.00 ± 1.15

After challenge	IW (g)	42.70 ± 1.15^c^	47.56 ± 0.77^ab^	51.10 ± 0.98^a^	45.23 ± 0.67^bc^	43.46 ± 1.06^bc^
	FW (g)	48.33 ± 1.25^d^	56.73 ± 0.66^ab^	60.93 ± 1.50^a^	53.36 ± 0.75^bc^	51.26 ± 0.63^cd^
	WG (g)	5.63 ± 0.31^b^	9.16 ± 0.81	9.83 ± 0.70	8.13 ± 0.66^ab^	7.80 ± 0.79^ab^
	FCR	2.58 ± 0.12^a^	1.63 ± 0.11^b^	1.52 ± 0.07^b^	1.76 ± 0.12^b^	1.90 ± 0.12^b^
	SGR (%d^−1^)	0.88 ± 0.04	1.25 ± 0.11	1.25 ± 0.07	1.18 ± 0.09	1.18 ± 0.13
	SR (%)	93.33 ± 1.66^a^	93.33 ± 1.66^a^	93.33 ± 3.33^a^	95.00 ± 2.88^a^	93.33 ± 1.66^a^

IW: initial weight; FW: final weight; WG: weight gain; FCR: feed conversion ratio; SGR: specific growth rate; SR: survival rate. *T*_1_ (control): nonsupplemented fish; *T*_2_: fish fed diet containing 1 × 10^6^ probiotic; *T*_3_: fish fed diet containing 1 × 10^8^ probiotic; *T*_4_: fish fed diet containing 0.5% hawthorn extract; *T*_5_: fish fed diet containing 1% hawthorn extract. Data represented as mean ± SE. Different letters in the same row indicate significant differences (*P* < 0.05).

**Table 4:** The activity of digestive enzymes in common carp, *Cyprinus carpio*, after 60 days of supplementation with experimental diets.

Parameters	*T*_1_ (control)	*T*_2_	*T*_3_	*T*_4_	*T*_5_
Amylase (U/mg protein)	1.10 ± 0.20^c^	2.50 ± 0.23^ab^	3.00 ± 0.17^a^	1.60 ± 0.22^bc^	1.53 ± 0.20^bc^
Protease (U/mg protein)	5.23 ± 0.50^c^	8.53 ± 0.50^a^	8.16 ± 0.61^ab^	7.03 ± 0.43^abc^	5.86 ± 0.52^bc^
Lipase (U/mg protein)	11.23 ± 0.67	11.90 ± 0.60	12.03 ± 0.57	11.33 ± 0.81	10.33 ± 0.52

*T*_1_ (control): nonsupplemented fish; *T*_2_: fish fed diet containing 1 × 10^6^ probiotic; *T*_3_: fish fed diet containing 1 × 10^8^ probiotic; *T*_4_: fish fed diet containing 0.5% hawthorn extract; *T*_5_: fish fed diet containing 1% hawthorn extract. Data represented as mean ± SE. Different letters in the same row indicate significant differences (*P* < 0.05).

**Table 5:** The serum immune parameters in common carp, *Cyprinus carpio*, after 60 days of supplementation with experimental diets.

Status	Parameters	*T*_1_ (control)	*T*_2_	*T*_3_	*T*_4_	*T*_5_
Before challenge	Lysozyme (U/ml)	27.33 ± 1.01^c^	36.26 ± 1.39^ab^	40.06 ± 0.97^a^	34.60 ± 1.15^b^	35.76 ± 0.86^ab^
	ACH_50_ (U/ml)	114.83 ± 3.44	115.56 ± 3.90	116.00 ± 3.32	123.03 ± 2.58	118.00 ± 3.78
	Total Ig (mg/dl)	18.90 ± 1.09	22.83 ± 1.01	21.86 ± 1.07	21.86 ± 1.04	22.90 ± 1.02
	NBT (OD at 540)	0.21 ± 0.01^b^	0.39 ± 0.03^a^	0.42 ± 0.03^a^	0.33 ± 0.04^ab^	0.34 ± 0.03^ab^
	MPO (OD at 450)	1.23 ± 0.28^c^	3.06 ± 0.26^ab^	3.50 ± 0.32^a^	2.56 ± 0.29^ab^	2.10 ± 0.20^bc^
	Protease (%)	4.43 ± 0.34^b^	4.73 ± 0.29^ab^	5.36 ± 0.40^ab^	5.53 ± 0.26^ab^	6.00 ± 0.28^a^

After challenge	Lysozyme (U/ml)	20.23 ± 0.95^b^	25.40 ± 1.06^a^	26.26 ± 0.72^a^	28.03 ± 0.83^a^	28.90 ± 0.97^a^
	ACH_50_ (U/ml)	104.50 ± 2.36^c^	107.66 ± 2.04^bc^	109.90 ± 1.59^abc^	118.70 ± 1.64^a^	115.00 ± 2.88^ab^
	Total Ig (mg/dl)	14.56 ± 0.82^b^	18.16 ± 0.72^ab^	18.60 ± 0.87^a^	18.20 ± 0.75^ab^	18.53 ± 0.72^a^
	NBT (OD at 540)	0.16 ± 0.01^b^	0.23 ± 0.00^ab^	0.25 ± 0.02^a^	0.21 ± 0.01^ab^	0.22 ± 0.01^ab^
	MPO (OD at 450)	1.03 ± 0.14^b^	1.60 ± 0.20^ab^	2.50 ± 0.34^a^	2.20 ± 0.17^a^	1.60 ± 0.20^ab^
	Protease (%)	2.36 ± 0.34	3.43 ± 0.40	3.65 ± 0.45	3.43 ± 0.47	3.40 ± 0.45

Total Ig: total immunoglobulin; ACH_50_: alternative complement activity; NBT: nitroblue tetrazolium; MPO: myeloperoxidase activity. *T*_1_ (control): nonsupplemented fish; *T*_2_: fish fed diet containing 1 × 10^6^ probiotic; *T*_3_: fish fed diet containing 1 × 10^8^ probiotic; *T*_4_: fish fed diet containing 0.5% hawthorn extract; *T*_5_: fish fed diet containing 1% hawthorn extract. Data represented as mean ± SE. Different letters in the same row indicate significant differences (*P* < 0.05).

**Table 6:** The liver metabolic enzymes in blood in common carp, *Cyprinus carpio*, after 60 days of supplementation with experimental diets.

Status	Parameters	*T*_1_ (control)	*T*_2_	*T*_3_	*T*_4_	*T*_5_
Before challenge	ALT (U/l)	24.83 ± 1.87^a^	23.16 ± 1.58^ab^	21.00 ± 1.73^ab^	18.50 ± 1.44^ab^	16.66 ± 1.20^b^
	AST (U/l)	93.33 ± 4.37^a^	88.33 ± 5.81^ab^	78.00 ± 4.93^ab^	75.66 ± 6.35^ab^	64.66 ± 5.20^b^
	ALP (U/l)	135.00 ± 8.66	127.66 ± 6.48	121.00 ± 7.81	117.00 ± 6.08	107.66 ± 5.36
	LDH (U/l)	586.00 ± 10.21^a^	553.33 ± 14.74^ab^	521.00 ± 11.59^b^	462.33 ± 10.39^c^	446.33 ± 14.83^c^

After challenge	ALT (U/l)	27.83 ± 1.87^a^	25.83 ± 1.64^ab^	24.33 ± 1.45^ab^	20.63 ± 2.58^ab^	17.66 ± 1.20^b^
	AST (U/l)	119.66 ± 4.09^a^	108.33 ± 3.75^ab^	97.33 ± 4.66^bc^	84.66 ± 4.80^cd^	73.33 ± 6.06^d^
	ALP (U/l)	160.66 ± 5.81^a^	144.33 ± 5.36^ab^	132.33 ± 7.21^bc^	127.00 ± 5.50^bc^	110.66 ± 4.97^c^
	LDH (U/l)	735.00 ± 13.22^a^	661.66 ± 17.05^b^	622.66 ± 11.85^b^	483.33 ± 41.52^c^	471.00 ± 18.00^c^

ALT: alanine aminotransferase; AST: aspartate aminotransferase; ALP: alkaline phosphatase; LDH: lactate dehydrogenase. *T*_1_ (control): nonsupplemented fish; *T*_2_: fish fed diet containing 1 × 10^6^ probiotic; *T*_3_: fish fed diet containing 1 × 10^8^ probiotic; *T*_4_: fish fed diet containing 0.5% hawthorn extract; *T*_5_: fish fed diet containing 1% hawthorn extract. Data represented as mean ± SE. Different letters in the same row indicate significant differences (*P* < 0.05).

**Table 7:** The serum biochemical profile in common carp, *Cyprinus carpio*, after 60 days of supplementation with experimental diets.

Status	Parameters	*T*_1_ (control)	*T*_2_	*T*_3_	*T*_4_	*T*_5_
Before challenge	Cortisol (ng/ml)	187.16 ± 3.94^a^	172.00 ± 4.04^ab^	168.66 ± 5.20^ab^	157.66 ± 4.33^b^	153.00 ± 5.29^b^
	Glucose (mg/dl)	79.00 ± 3.21	75.50 ± 3.17	71.33 ± 2.60	77.00 ± 3.78	71.33 ± 2.40
	TP (g/dl)	2.95 ± 0.13^b^	3.33 ± 0.09^ab^	3.32 ± 0.14^ab^	3.89 ± 0.22^a^	3.82 ± 0.14^a^
	Globulin (g/dl)	1.65 ± 0.13^b^	2.00 ± 0.05^ab^	1.94 ± 0.04^ab^	2.21 ± 0.14^a^	2.16 ± 0.12^a^
	Albumin (g/dl)	1.30 ± 0.05^c^	1.33 ± 0.04^bc^	1.38 ± 0.10^abc^	1.68 ± 0.09^a^	1.66 ± 0.03^ab^

After challenge	Cortisol (ng/ml)	207.66 ± 4.05^a^	193.66 ± 4.40^ab^	185.66 ± 4.63^b^	165.00 ± 2.88^c^	157.00 ± 3.60^c^
	Glucose (mg/dl)	88.33 ± 2.60^a^	82.66 ± 2.90^ab^	84.66 ± 2.72^ab^	80.00 ± 1.73^ab^	75.66 ± 2.33^b^
	TP (g/dl)	2.77 ± 0.13^b^	3.03 ± 0.09^ab^	3.19 ± 0.11^ab^	3.53 ± 0.19^a^	3.47 ± 0.16^a^
	Globulin (g/dl)	1.57 ± 0.12	1.77 ± 0.06	1.68 ± 0.06	1.93 ± 0.06	1.98 ± 0.10
	Albumin (g/dl)	1.20 ± 0.05	1.26 ± 0.05	1.50 ± 0.16	1.59 ± 0.15	1.49 ± 0.05

TP: total protein. *T*_1_ (control): nonsupplemented fish; *T*_2_: fish fed diet containing 1 × 10^6^ probiotic; *T*_3_: fish fed diet containing 1 × 10^8^ probiotic; *T*_4_: fish fed diet containing 0.5% hawthorn extract; *T*_5_: fish fed diet containing 1% hawthorn extract. Data represented as mean ± SE. Different letters in the same row indicate significant differences (*P* < 0.05).

**Table 8:** The serum antioxidant enzymes in common carp, *Cyprinus carpio*, after 60 days of supplementation with experimental diets.

Status	Parameters	*T*_1_ (control)	*T*_2_	*T*_3_	*T*_4_	*T*_5_
Before challenge	MDA (*μ*mol/l)	50.23 ± 2.22	48.56 ± 1.84	48.30 ± 2.04	45.43 ± 2.52	42.23 ± 1.30
	SOD (U/ml)	30.43 ± 0.80^b^	32.40 ± 0.94^ab^	34.36 ± 0.74*a*	35.96 ± 0.72^a^	36.13 ± 0.80^a^
	CAT (U/ml)	112.16 ± 3.60^a^	113.16 ± 2.89^a^	115.00 ± 2.68^a^	114.33 ± 4.33^a^	115.66 ± 3.32^a^
	GPx (U/ml)	150.03 ± 2.56^b^	155.00 ± 2.88^ab^	157.16 ± 3.60^ab^	160.00 ± 2.02^ab^	165.00 ± 1.73^a^

After challenge	MDA (*μ*mol/l)	61.23 ± 2.91^a^	56.16 ± 2.36^ab^	56.23 ± 2.40^ab^	49.60 ± 2.26^bc^	43.90 ± 1.37^c^
	SOD (U/ml)	29.70 ± 0.95^c^	32.90 ± 1.06^bc^	34.53 ± 0.89^ab^	36.80 ± 1.04^ab^	37.50 ± 0.68^a^
	CAT (U/ml)	110.26 ± 3.42	110.50 ± 2.59	113.06 ± 2.73	113.16 ± 4.18	116.80 ± 3.34
	GPx (U/ml)	145.83 ± 2.51^c^	152.43 ± 2.69^bc^	155.23 ± 3.03^abc^	160.00 ± 3.88^ab^	167.33 ± 1.76^a^

MDA: malondialdehyde activity; SOD: superoxide dismutase activity; CAT: catalase activity; GPx: glutathione peroxidase activity. *T*_1_ (control): nonsupplemented fish; *T*_2_: fish fed diet containing 1 × 10^6^ probiotic; *T*_3_: fish fed diet containing 1 × 10^8^ probiotic; *T*_4_: fish fed diet containing 0.5% hawthorn extract; *T*_5_: fish fed diet containing 1% hawthorn extract. Data represented as mean ± SE. Different letters in the same row indicate significant differences (*P* < 0.05).

## Data Availability

The datasets generated during and/or analysed during the current study are available from the corresponding author on reasonable request.

## Supplementary Materials

Supplementary Materials The file contains Supplementary Figure 2 with legends.
