# Supplementary material for: The Probiotic Lactobacillus sakei Subsp. Sakei and Hawthorn Extract Supplements Improved Growth Performance, Digestive Enzymes, Immunity, and Resistance to the Pesticide Acetamiprid in Common Carp (Cyprinus carpio)
Source: Aquac Nutr. 2023 Mar 6;2023:8506738. doi: 10.1155/2023/8506738 (PMC10010885; doi:10.1155/2023/8506738)
Supplement: Supplementary Materials — The file contains Supplementary Figure 2 with legends. [file 8506738.f1.docx]

**B**

**Fig. 2.** The intestinal lactic acid bacteria load in common carp, *Cyprinus carpio* after 60 days supplementation with experimental diets: T_1_ (control): non-supplemented fish, T_2_: fish fed diet containg 1 × 10^6^ probiotic, T_3_: fish fed diet containg 1 × 10^8^ probiotic, T_4_: fish fed diet containg 0.5 % hawthorn extract, T_5_: fish fed diet containg 1 % hawthorn extract. LAB: lactic acid bacteria concentration, TBC: Total bacterial count. Data represent as mean ± SE. Different letters in the same row indicate significant differences (P<0.05).
